# Comparative Analysis of the Chemical Composition and Physicochemical Properties of the Mucilage Extracted from Fresh and Dehydrated *Opuntia ficus indica* Cladodes

**DOI:** 10.3390/foods10092137

**Published:** 2021-09-10

**Authors:** Michelle Quintero-García, Elsa Gutiérrez-Cortez, Moustapha Bah, Alejandra Rojas-Molina, María de los Angeles Cornejo-Villegas, Alicia Del Real, Isela Rojas-Molina

**Affiliations:** 1 Laboratorio de Investigación Química y Farmacológica de Productos Naturales, Facultad de Química, Universidad Autónoma de Querétaro, Cerro de las Campanas S/N, Centro Universitario, Santiago de Querétaro C.P. 76010, Mexico; adr_mich@hotmail.com (M.Q.-G.); moubah@uaq.mx (M.B.); rojasa@uaq.mx (A.R.-M.); 2 Doctorado en Ciencias Químico-Biológicas, Facultad de Química, Universidad Autónoma de Querétaro, Cerro de las Campanas S/N, Centro Universitario, Santiago de Querétaro C.P. 76010, Mexico; 3 Laboratorio de Procesos de Transformación y Tecnologías Emergentes de Alimentos, Departamento de Ingeniería y Tecnología, FES-Cuautitlán, Universidad Nacional Autónoma de México, Cuautitlán Izcalli C.P. 54714, Mexico; angiecornejo@unam.mx; 4 Centro de Física Aplicada y Tecnología Avanzada, Universidad Nacional Autónoma de México, Juriquilla C.P. 76230, Mexico; adelreal@unam.mx

**Keywords:** mucilage, *O. ficus indica*, mechanical separation, separation efficiency, sustainable extraction, physicochemical properties

## Abstract

The development of sustainable extraction methods to obtain natural products constitutes a challenge for the food industry. The aim of this work was to compare yield, separation efficiency, chemical composition, and physicochemical properties of the mucilage extracted from fresh cladodes (FNM) and mucilage extracted from dehydrated cladodes (DNM) of *O. ficus indica*. Suspensions of fresh and dehydrated cladodes (4% *w*/*w*) were prepared for mucilage extraction by using a mechanical separation process. Subsequently, the separated mucilage was precipitated with ethyl alcohol (1:2 *v*/*v*) then, yield and separation efficiency were determined. The mucilage was characterized by measuring Z potential, viscosity, color, and texture attributes. Additionally, chemical proximate analysis, scanning electron microscopy, and thermogravimetric analysis (TGA) were conducted. No significant differences (*p* < 0.05) were detected in the yield and separation efficiencies between samples. Nevertheless, the dehydration process of cladodes prior to mucilage extraction increased protein, ashes, nitrogen free extract, and calcium content. The viscosity was higher in DNM than in FNM. The TGA revealed a different thermal behavior between samples. In addition, the DNM showed lower L (darkness/lightness), cohesiveness, adhesiveness, and springiness values than those of FNM. These results support that differences found between the chemical and physicochemical properties of DNM and those of FNM will determine the applications of the mucilage obtained from the *O. ficus indica* cladodes in the food, pharmaceutical, and cosmetic industries.

## 1. Introduction

The *Opuntia ficus indica* cladode is a common vegetable in the traditional diet of the Mexican population, which reaches its maximum commercial value when it weighs less than 150 g [1]. Cladodes weighing more than 200 g are not eaten fresh due to their extremely fibrous texture; for this reason, it is preferred to dehydrate and grind them to obtain granular solids [2].

The cladode of this species is a food that provides numerous health benefits, including: protection against free radicals and cancer, attributed to the presence of antioxidant components; reduction of serum glucose and cholesterol levels; and as a co-adjuvant to control weight due to its low content of lipids and carbohydrates [3,4,5]. Furthermore, *O. ficus indica* cladodes constitute an important source of minerals in the diet, mainly calcium [6]. In fact, our research group has previously demonstrated, employing models of growing and ovariectomized rats, that calcium in *O. ficus indica* is bioavailable [7,8,9]. The content of water in these cladodes is high (90–96 g/100 g), thus dehydration constitutes an alternative to increase the shelf life of this product, reducing transportation and storage expenses [10].

The mucilage obtained from *O. ficus indica* is a viscous biopolymer, whose main monomeric units are D-glucose, D-galactose, L-arabinose, D-xylose, L-rhamnose, D-galacturonic acid, and glucuronic acid. Consequently, mucilage constitutes a fraction of the soluble dietary fiber of this cactus [3]. The age of the cladodes is not a restriction for obtaining mucilage, which is analogous to gums and represents a material of interest for the industrial sector, due to both, its physicochemical properties, and its health benefits [11]. This biopolymer has been applied in the pharmaceutical, cosmetic, medical, and food areas due to its physicochemical and rheological properties, emulsifying capacity, as an auxiliary in gels formation, as well as in the elaboration of edible films and coatings [12,13,14]. Various methodologies have been reported for extracting the mucilage from fresh cladodes, using different process conditions, such as temperature, extraction time, and different extraction solvents. It should be noted that results regarding mucilage yield obtained in different investigations are very dissimilar, most likely as a result of variations between the extraction procedures and the maturity stage and variety of the cladodes. Up to now, the efficiency of the extraction process of mucilage from *O. ficus indica* has not been reported [15,16]. Moreover, although Contreras-Padilla et al. [17] compared the rheological properties of the mucilage extracted from *O. ficus indica* cladodes at different maturity stages, currently, a comparative analysis of the physicochemical properties of the mucilage extracted from fresh and dehydrated cladodes has been scarcely described.

In the present research, we hypothesized that physicochemical properties of the mucilage obtained from dehydrated cladodes are different from those of the mucilage extracted from fresh cladodes, which have an important impact on the applications that mucilage could have, mainly in the food industry [18]. The purpose of this work was to compare the chemical composition, physicochemical properties, and microstructure of the mucilage extracted from fresh *O. ficus indica* cladodes with those of the mucilage obtained from dehydrated cladodes, by using a mechanical separation process by centrifugation in order to determine whether the dehydration process affects the properties of mucilage and, therefore its applications in the food industry. In addition, this study proposes an environmentally friendly method to obtain mucilage from *O. ficus indica* cladodes.

## 2. Materials and Methods

### 2.1. Vegetal Material

*Opuntia ficus indica* cladodes with an average weight of 400 g (100 days after sprouting) were cultivated with an organic fertilizer and collected during the summer of 2019 in the experimental field of the Engineering Department of the Autonomous University of Querétaro.

### 2.2. Mucilage Extraction from O. ficus indica Cladodes

The *O. ficus indica* cladodes were washed and both, spines and crown were manually removed. During the drying process, the cladodes were placed on stainless steel trays to dry in a forced air oven (EPS, Mod. SM05, Santa Ana, CA, USA). The drying temperature was 70 °C with an air flow of 1.4 m/s. The moisture content was determined in the dry material, and afterward, it was ground using a hammer mill (PULVEX 200, Mexico City, Mexico) with a mesh size 0.8 mm. Subsequently, the solids were passed through a No. 60 sieve (USA series) to obtain solids with homogeneous size. On the other hand, the fresh cladodes were wet ground in a blade mill (RETSCH, Mod. GM 300, Newton, PA, USA), with a mesh size of 0.8 mm, in order to obtain a suspension that passed through a No. 60 sieve (USA series).

Thereafter, two suspensions were prepared. One of them was obtained from the fresh ground cladodes and the moisture content was analyzed by the method 925.10 of the Association of Official Analytical Chemists (AOAC, 2000) [19] to determine the content of total solids.

Another suspension was prepared with the granular solids obtained from dehydrated cladodes and water, using a homogenizer (IKA-WERKE, Mod. Eurostar BSC.S1, Wilmington, NC, USA) at a speed of 450 rpm for 5 min. Both suspensions were stored 4 h at 10 °C. Thereafter, the resulting suspension was centrifuged to separate the mucilage in a disc stack centrifuge (DIDACTA, Mod. TAG1/d, Torino, TO, Italy) with a feeding speed of 200 mL/min and a centrifugation rate of 7000 rpm [6]. The light fraction, containing the soluble fiber, was separated from the heavy fraction (insoluble fiber). Mucilage was obtained from the light fraction by precipitation with ethyl alcohol 96% (*v*/*v*) in a 1:2 (*v*/*v*) ratio (light fraction: ethyl alcohol). Subsequently, the mucilage was vacuum filtered on a Büchner funnel at 5.33 kPa using a 20 µm pore size polyester-cotton filter (70–30%). Finally, the mucilage was dehydrated in a forced air oven (EPS, Mod. SM05, Santa Ana, CA, USA) at 35 °C during 40 min. The suspension obtained from fresh cladodes was processed employing the same conditions just described. *Mucilage yield* extracted from fresh and dehydrated cladodes was obtained by weighting total solids in mucilage and calculating the percentage based on the weight of total solids in 100 g of cladodes, employing the Equation (1) [20].

(1) $$Mucialge\;yield\;\left(\%\right)=\left(\frac{Wm}{Wc}\;\right)\times 100$$

where:

*Wm =* Weight of total solids in the mucilage of *O. ficus indica* cladodes (g)

*Wc =* Weight of total solids in *O. ficus indica* cladodes (g)

The separation efficiency was calculated using the turbidity values according to the Equation (2) reported by Bai et al. [21] with some modifications. This was proposed considering the turbidity of the suspensions and the turbidity of the centrifuged samples (light fraction) at the processing conditions. The turbidity was analyzed with a turbidimeter (HANNA INSTRUMENTS, Mod. LP 2000, Woonsocket, RI, USA) and the method 1889-88a [22].

(2) $$Separation\;efficiency\;\left(\%\right)=\left(\left(Tsn-Tlf\right)/Tsn\right)\;\times 100$$

where:

*Tsn =* Turbidity of suspension obtained with fresh and dehydrated cladodes (ntu)

*Tlf =* Turbidity of light fraction (ntu)

### 2.3. Proximal Chemical Analysis

Fresh and dehydrated cladodes, as well as their respective mucilages were analyzed according to the methods established by the American Association of Cereal Chemists (AACC, 2000) [23] and the AOAC (2000) [19] and the nitrogen free extract (NFE) was calculated by difference (% NFE = 100 − [% moisture + % Crude fiber + % Crude protein + % Ether extract + % Ash]).

### 2.4. Calcium Content Analysis

The calcium content in mucilage samples was determined by the dry ashing method (968.08) [19] with an atomic absorption spectrophotometer (Perkin Elmer, Mod. Analyst 300, Boston, MA, USA) and a flame detector. The operating conditions were reported by Rojas-Molina et al. [6].

### 2.5. Morphological Characterization of O. ficus indica Cladodes and Mucilage Obtained from Fresh and Dehydrated Cladodes

The morphology of *O. ficus indica* cladodes and the mucilage extracted from fresh and dehydrated cladodes was studied by low vacuum scanning electron microscopy (LV-SEM) using a JSM 5600LV (Tokyo, Japan) microscope (5 nm resolution), equipped with an energy dispersive X-ray spectrometer (Noran instrument, Mod. Voyager 4.2.3). The analysis conditions were those reported by Mendoza-Ávila et al. [24].

### 2.6. Characterization of the Mucilage Extracted from Fresh and Dehydrated O. ficus indica Cladodes by Fourier Transform Infrared (FT-IR) Analysis

Mucilage samples were characterized by using an FTIR spectrophotometer (Perkin Elmer, Mod. Spectrum Two, Boston, MA, USA) with ATR (Attenuated Total Reflectance).

### 2.7. Z Potential

Mucilage powders obtained from fresh and dehydrated cladodes were dissolved in deionized water to obtain mucilage solutions of 0.1% (*w*/*v*) at 40 °C. The solids were mixed with a homogenizer at 600 rpm for 30 min (IKA-WERKE, Mod. Eurostar BSC.S1, Wilmington, NC, USA). The dispersions were stored for 24 h until analysis. Z potential assessments were conducted using a Zetasizer Nano ZS90 equipment (Malvern Instruments Ltd., Malvern, Worcestershire, UK) at a temperature of 25 °C to obtain the Z potential and the particle size.

### 2.8. Viscosity of the Mucilage Extracted from Fresh and Dehydrated O. ficus indica Cladodes

Mucilage powders obtained from fresh and dehydrated cladodes were dissolved in distilled water to prepare mucilage solutions at different concentrations of 1.0, 1.5, 2.0, and 2.5% (*w*/*v*) at 25 °C with magnetic stirring for 45 min to complete the hydration. Then, the solutions were cooled at room temperature for analysis. Measurements were performed in rotational mode at different shear rates in a range from 64.6 to 600 s^−1^ in a viscosimeter with concentric cylinder geometry (Mettler-Toledo, Mod. RM180 Rheomat, Columbus, OH, USA) and a rotating cylinder sensor (DIN 1). Data obtained from this analysis were used to obtain the graphs of shear straining (Pa) versus shear rate (s^−1^) and the rheograms of viscosity (Pa.s) versus shear rate (s^−1^). The flow behavior index (*n*) and consistency index (*k*) values were computed by fitting the power law model [25].

### 2.9. Thermogravimetric Analysis (TGA)

Thermal gravimetric analyses of the samples were carried out with a TGA Q5000IR equipment (TA Instruments, New Castle, DE, USA). A nitrogen atmosphere was used at a flow rate of 30 mL/min, a heating rate of 10 °C/min, temperature ranging from 25 to 600 °C and analysis was carried out by employing the TRIOS 4.3.0. 38388 software. All measurements were performed in triplicate.

### 2.10. Color Measurement

Color measurement of the mucilage samples was performed using a colorimeter (Minolta, CR300, Tokyo, Japan) and the granular solids device. Three-dimensional scales expressed as Hunter L, a, and b were used to quantify color values, which were recorded as L, darkness/lightness (0, black; 100, white); a (−a, greenness; +a, redness); and b (−b, blueness; +b, yellowness). Additionally, the total color difference (ΔE) was calculated according to Equation (3). Color measurements were repeated from three different positions [26].

(3) $$\Delta E=\frac{{\left({\left(L*1-L*2\right)}^{2}+{\left(a*1-a*2\right)}^{2}+{\left(b*1-b*2\right)}^{2}\right)}^{1}}{2}$$

### 2.11. Texture Profile Analysis (TPA)

The samples were dissolved in distilled water at 35 °C to prepare 4% (*w*/*v*) solutions using a thermo shaker (Thermo Scientific, Mod. 4625Q, Waltham, MA, USA) at a maximum rate of 250 rpm. Then, samples were stored at room temperature for 24 h until analysis. A universal texturometer (Shimadzu, Mod. EZ-S, Kioto, Japan) was used to obtain force-time curves of a two-cycle compression test. All experiments were conducted under a controlled temperature of 25 °C. A cylinder of 2 cm in diameter compressed the samples placed in a Petri dish (diameter 6.0 cm, height 1.5 cm). The cylinder was allowed to descend at a rate of 1.3 mm/s to a compression depth of 8 mm (20% compression). Once the compression stroke was completed, the direction of the cylinder was reversed and started its upward stroke. Then, a second down and up cycle was run on the same sample with a 5 s pause before the second compression cycle. The instrument automatically recorded the force–time curve. Five replicates were conducted for each sample.

### 2.12. Statistical Analysis

The results obtained from the physicochemical characterization of the mucilage samples were analyzed with Student’s *t*-test with an α value of 0.05. In all cases, the statistical package SPSS version 2.2 was used.

## 3. Results and Discussion

### 3.1. Yield and Separation Efficiency of the Mucilage Extracted from Fresh and Dried O. ficus indica Cladodes

The content of total solids of the fresh ground cladodes was 4% (*w*/*w*). This determination was carried out with the purpose of ensuring the same total solids content in the suspension of the dehydrated cladodes.

Yields obtained for the mucilage extraction from dehydrated and fresh *O. ficus indica* cladodes were 15.69 ± 0.04 and 13.06 ± 0.19 g/100 g respectively. No significant differences were observed between both values (*p* ≤ 0.05). These results are in accordance with Contreras-Padilla et al. [17], who used similar conditions for extracting the mucilage from cladodes of this plant species. However, in other studies higher yields of mucilage, ranging from 16.50 to 33%, have been reported [27,28,29]. These discrepancies can be attributed to various factors including: (1) the type of solvent used in the extraction process, the polarity of which significantly influences the solubility and mass transfer characteristics of the compounds to be extracted [30]; (2) cladodes maturity stage, since a direct relationship between mucilage yield and stalk age has been observed; and (3) temperature and time of extraction; It has been previously demonstrated that young cladodes possess a high soluble fiber content which produces higher yields of mucilage, whereas, as cladodes get older, the insoluble fiber content increases [31]. Furthermore, time and extraction temperature are critical, since they involve heat energy transfer, by convection and conduction, from the solvent to the nucleus of the matrix particles, as well as solvent interaction with target particles [32].

Although mucilage extraction yields have been described by some authors [17,27,28,29], there is little information related to the efficiency of this process. In this study, the efficiency of mucilage extraction from fresh and dehydrated cladodes was 96.7 ± 0.06 and 97 ± 0.01%, respectively. These results indicate that dehydration of the cladodes does not affect the efficiency of hydrocolloid separation. The process that we employed to obtain the mucilage from *O. ficus indica* cladodes involved a centrifugation step to separate insoluble and soluble fiber, the latter containing the mucilage. Centrifugation allows separation of impurities from hydrocolloids used for food applications, such as those from macroalgae [33]. Thus, it is likely that the *O. ficus indica* mucilage obtained by this extraction process could be used for food and pharmaceutical applications [34].

The use of ethanol, which is an eco-friendly solvent, to extract the mucilage from *O. ficus indica* cladodes represents one of the advantages of this extraction method. According to the environmental health and safety guidelines, the danger that ethanol represents for health and the environment is relatively low (less than 3.0 points) [35]. Other advantages offered by this method are that it does not require heating and the extraction time is short compared to other techniques. Moreover, in the present work cladodes at a late maturity stage, which are considered as waste and have no commercial value, were used. Therefore, this is a sustainable extraction method [36].

### 3.2. Chemical Composition of Mucilage Extracted from Fresh and Dehydrated Cladodes

The proximal chemical analysis and calcium content of the mucilage extracted from fresh (FNM) and dehydrated cladodes (DNM) are summarized in Table 1. Significant differences (*p* < 0.05) were detected in protein, ashes, nitrogen free extract (NFE) and calcium contents.

The moisture content in the mucilage extracted from fresh and dehydrated cladodes showed values around 4%. This percentage is similar to that previously reported (4.4–4.5%), when a flow spray dryer with an inlet air temperature at 170 °C was used to dehydrate the *O. ficus indica* mucilage [37]. It has been reported that the use of high temperature for relatively long periods of time is required to remove excess water from the mucilage, which is composed mainly of long-chain acid heteropolysaccharides that absorb and retain a high water content [38].

In the present study, the protein content of both mucilage samples was higher than that obtained by Toit et al. (2018) [39] (2.7–3.2 g/100 g) and similar to that observed by Gebresamuel and Gebre-Mariam [40] (6.82 g/100 g) in mucilage extracts of *Opuntia* spp. cladodes. While the lipid content that we found in the mucilage samples was higher than that reported by the two previously mentioned research groups, who found lipid concentrations between 0.4 and 0.9% [39,40]. In addition, long chain fatty acids (α-linolenic and linoleic acid, among others) were found in *O. ficus indica* cladodes [39]. These fatty acids are highly resistant to thermal hydrolysis at temperatures between 90 and 160 °C for periods of time between 30 min and 8 h [41]. This implies that the temperature used in this study to carry out the dehydration of *O. ficus indica* cladodes does not affect the integrity of the fatty acids they contain. On the other hand, no significant differences (*p* < 0.05) were detected in the crude fiber content between the samples (see Table 1), these results may be associated with the efficiency of mucilage extraction from fresh and dehydrated cladodes, which were similar. The crude fiber content that we detected in FNM and DNM was lower than that reported by Sepúlveda et al. [27] (0.69%) and higher than that found by Toit et al. [39] and Gebresamuel and Gebre-Mariam [40] (0.2 and 0.06%, respectively). It has been proposed that *O. ficus indica* mucilage is constituted by different water-soluble fractions: (1) a pectin with calcium dependent gelling properties, (2) a non-gelling mucilage, and (3) a polysaccharide fraction with thickening properties representing less than 10% of the water-soluble material [42,43]. Therefore, low crude fiber content was expected, since in the crude fiber analysis only water insoluble components, such as cellulose, hemicellulose, and lignin are quantified, [44]. Differences observed in proteins, ashes, NFE, and calcium contents of the samples studied in this research, with respect to samples analyzed in other investigations can be attributed to several factors, such as: variation in the extraction process, the age of the cladodes, the growing season, and edaphic conditions. Undoubtedly these factors influence the nutrient content of the cladodes from this species [27,29,39,45].

The calcium content in the mucilage obtained from dehydrated cladodes (19.05 ± 0.25 mg/g) was significantly higher (*p* < 0.05) than that found in the mucilage extracted from fresh cladodes (15.18 ± 0.15 mg/g). Both values are similar to those observed by Monrroy et al. [28]. Nevertheless, differences can be attributed to different edapho-climatic cultivar conditions and cladode age [46]. Most of the calcium contained in the mucilage is in the form of CaCO_3_, which is bioavailable [6,8,9]. As expected, due to water loss, calcium, ash, protein, and NFE contents were significantly higher in mucilage obtained from dehydrated cladodes. It is important to mention that the higher protein content in mucilage increases its capacity as an emulsifying agent, promoting the formation of an oil-in-water emulsion, which is stabilized by the proteins [47]. Due to the fact that the carbohydrate (NFE) content was determined by difference, evidently the amount of this component was higher in the mucilage extracted from fresh cladodes.

### 3.3. Morphology of Mucilage Extracted from Fresh and Dehydrated Cladodes

**Figure 1:**
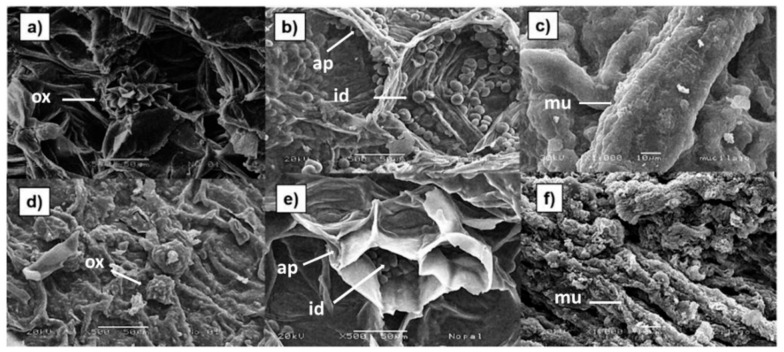
Scanning electron microscopy (SEM) images of fresh (**a**,**b**) and dehydrated (**d**,**e**) *O. ficus indica* cladodes and mucilage extracted from fresh (**c**) and dehydrated (**f**) cladodes. (**a**) Calcium oxalate crystals (ox) in fresh cladodes, (**d**) calcium oxalate crystals (ox) in dehydrated cladodes, (**b**) aqueous parenchyma (ap) in fresh cladodes with mucilage-secreting idioblasts inside (id), (**e**) aqueous parenchyma (ap) in dehydrated cladodes with mucilage-secreting idioblasts inside (id), (**c**) mucilage (mu) extracted from fresh cladodes, (**f**) mucilage (mu)extracted from dehydrated cladodes.

Figure 1 shows micrographs of fresh (Figure 1a,b) and dehydrated (Figure 1d,e) *O. ficus indica* cladodes. Figure 1a,d show the presence of calcium oxalate crystals in the insoluble fiber, both in fresh and dehydrated cladodes; these crystals have been identified by other authors too [6,17]. Calcium oxalate crystals are formed within cells known as crystalline idioblasts, whose function is to maintain an ionic balance and regulate the osmotic pressure of the plant [48]. In Figure 1b,e the internal parenchyma of fresh and dehydrated cladodes is observed. This tissue is characterized by the presence of isodiametric cells, which possess thin cell walls made up mainly of cellulose, and whose function is to store water, for this reason, this tissue is also known as aqueous parenchyma or hydrenchyma [49]. Inside the aqueous parenchyma, the presence of spherical and globose structures known as mucilage cells or mucilage-secreting idioblasts, which are characteristic of *Opuntia* species, can be distinguished (see arrows). The main function of these cells is the retention of water when the plant is exposed to long periods of drought. In addition, these cells are specialized to retain more than 30% of water as a reserve in the parenchyma area [49,50,51,52]. It is worth noting that size of the cells of the internal parenchyma and the mucilage-secreting idioblasts of the dehydrated cladodes (Figure 1e) is smaller than that of the fresh cladodes (Figure 1b). In addition, it is evident that the mucilage-secreting idioblasts have lost their spherical shape (Figure 1b). This can be explained by the removal of water from the dehydrated cladodes during drying. It has been reported that mucilage-secreting idioblasts can represent up to 14% of the cladode dry weight.

Figure 1c,f show mucilage obtained from fresh and dehydrated cladodes, respectively. In cactus stems, mucilage together with gums and pectin are constituents of soluble fiber and constitute a colloidal system that prevents dehydration of plant tissues [53,54]. Bayar et al. [55] demonstrated that 50% of mucilage composition is pectin; nevertheless, mucilage showed 28% more water holding capacity than pectin. This explains why mucilage retains more than 30% of the total stored water in parenchyma [54]. Micrographs of Figure 1c,f show that the mucilage has an elongated structure, which differs from what was reported by León-Martinez et al. [37], who observed that powdered mucilage was composed of agglomerated semi-spherical particles. These authors possibly observed clumps of mucilage cells (mucilage-secreting idioblasts) as a result of the mucilage extraction process that they used, which consisted of a decoction and stirring. In the case of the present study, mechanical separation of the mucilage at high speed most likely provokes idioblasts’ disruption, which consequently causes the release of mucilage from the cells, therefore no globular structures are observed. Regarding this, it has been reported that disc stack centrifugation promotes yeasts and algal cell disruption leading to the release of various cell components, including lipids [56,57]. It is important to mention that various clusters of particles are observed on the surfaces of the elongated structures of the mucilage. These accumulations are more abundant in the mucilage obtained from dehydrated cladodes and possibly correspond to mineral salts, mainly calcium carbonate. The presence of this crystalline compound in *O. ficus indica* soluble fiber has been previously reported [6].

### 3.4. FTIR Spectral Characterization of the Mucilage Extracted from Cladodes

**Figure 2:**
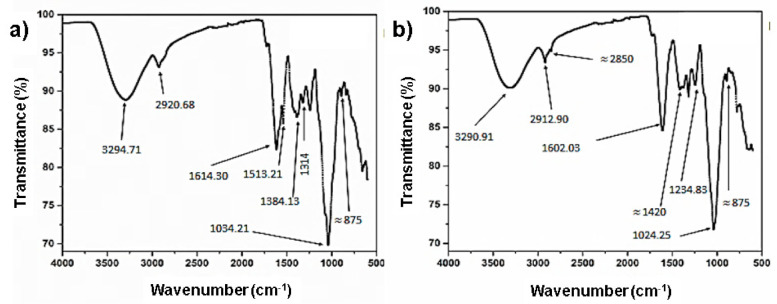
(**a**) FTIR spectrum of mucilage extracted from fresh *Opuntia ficus indica* cladodes. (**b**) FTIR spectrum of mucilage extracted from dehydrated *O. ficus indica* cladodes.

The IR spectra of the mucilage extracted from fresh and dried *O. ficus indica* cladodes showed differences in both the position and intensity of some bands (Figure 2a,b). A slight shift towards a lower frequency was observed in the characteristic broad band corresponding to OH-stretching vibration, which appeared at 3294 cm^−1^ in the mucilage obtained from fresh cladodes and 3290 cm^−1^ in the spectrum of mucilage obtained from dehydrated cladodes (see arrows). Additionally, this band showed lower intensity. This could be attributed to the loss of some of the hydrogen bonds formed between water, which is abundant in the fresh cladodes, and hydroxyl groups of carbohydrates and acids. The same tendency was observed for the ν_as_ of CH_2_ groups, which decreased from 2920 to 2912 cm^−1^ in the mucilage obtained from dehydrated cladodes (see arrows). This band corresponds to the stretching vibration of hydroxymethylene groups present both in the pyranose and furanose conformations of galactose, arabinose, and xylose, which are the most frequent monosaccharides identified in *Opuntia* mucilage. In fact, the viscosity of mucilage has been mainly attributed to the high content of arabinose [16]. On the other hand, a decrease in frequency was also observed for the band corresponding to the carboxylate group (COO^−^), which appears at approximately 1600 cm^−1^ (strong ν_as_). In the case of the spectrum of the mucilage obtained from fresh cladodes, this band splits into a second weaker band at 1513 cm^−1^ (ν_s_ COO^−^), as a result of the H bonds established between acids contained in the mucilage and water. It is very likely that these carboxylate groups belong to uronic acid, which is commonly found in mucilage [28]. It has been demonstrated that the carboxylate group of uronic acid interacts with water and cationic ions, such as calcium, which makes an important contribution to the viscosity of mucilage [16]. Weak bands at 1420 cm^−1^ (overlapped in mucilage extracted from fresh cladodes and more visible in mucilage extracted from dehydrated cladodes) and 875 cm^−1^ in both mucilage samples (see arrows) indicated the presence of CO_3_^2−^. Finally, a decrease (from 1034.21 cm^−1^ to 1024.25 cm^−1^) in the ν of the band corresponding to the C-O bond of sugar units was also observed in the case of the mucilage extracted from dehydrated cladodes, as a consequence of the loss of water. It is worth mentioning that dry mucilage is the most suitable material for a longer shelf life of *Opuntia* products before reconstitution for human consumption.

### 3.5. Z Potential and Particle Size

The zeta potential is the overall charge a particle acquires in a specific medium and shows the degree of repulsion between charged particles in the dispersion. Thus, this parameter is used to measure the stability of a colloid solution as a function of the molecules’ charges dispersed in the system [58]. No statistically significant differences *(p* ≤ 0.05) were observed in the Z potential in the dispersions of mucilage extracted from fresh and dehydrated cladodes (−21.73 ± 2.38 and −19.30 ± 1.31 mV, respectively). These values are within the range reported by Quinzio et al. [59] for the mucilage extracted from *O. ficus indica* (from −1.0 to −32 mV) with pH values from 2.0 to 10.0. The variations between the Z potential values are attributed to the presence of the carboxyl group of galacturonic acid in the mucilage. At a basic pH, these carboxyl groups ionize to give rise to carboxylates, resulting in an increase in the negative charge of the Z potential [60]. Accordingly, negative values of Z potential have been reported for several biopolymers in a pH range from 2.0 to 9.0, including pectin (−15 mv to 25 mV) [61], sodium alginate (−8.7 mV to −68.4 mV) [62], Arabic gum (−2 mV to −21 mV), beet pectin (−2.5 mV to −33 mV), and corn fiber gum (−3 mV to −19 mV) [63].

No statistical difference (*p* ≤ 0.05) was found between the particle size values obtained from the mucilage extracted from fresh cladodes (2623 ± 52.93 nm) and those of the mucilage extracted from dried cladodes (2672 ± 42.31 nm). These values are in accordance with what was reported by Cortés-Camargo et al. [60] for the particle size of the *O. ficus indica* mucilage (1.64 to 2.52 μm). The particle sizes found in the *O. ficus indica* mucilage were smaller than those of arabic gum (37 to 790 μm) [64], jackfruit pectin (363.6 μm) [65], and guar gum (46.4–315 μm) [66].

Similar Z potential and particle size values found in the mucilage extracted from fresh and dehydrated cladodes implies that the dehydration process of the cladodes prior to mucilage extraction does not modify these physicochemical properties of the hydrocolloid. On the other hand, the particle size and negative Z potential values detected in mucilage extracted from fresh and dehydrated cladodes indicate that this hydrocolloid can interact with polycationic biopolymers such as proteins, to obtain microspheres through the complex coacervation method. These microstructures can be applied in the food and pharmaceutical industries for the encapsulation of biologically active compounds to obtain controlled delivery systems [67].

### 3.6. Viscosity of the Mucilage Extracted from Fresh and Dehydrated Cladodes

**Figure 3:**
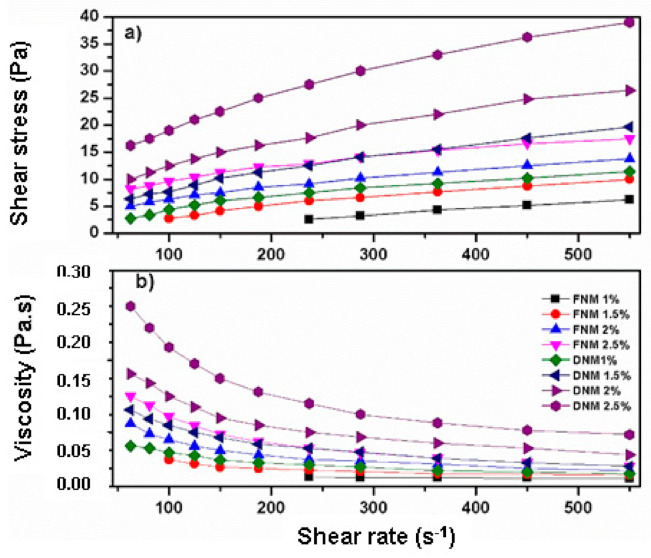
Shear strain (**a**) and viscosity (**b**) values versus shear rate of mucilage extracted from fresh cladodes (FNM) and mucilage extracted form dehydrated cladodes (DNM) at different concentrations (1, 1.5, 2 and 2.5% *w*/*v*).

The viscosity and shear straining values versus shear rate of samples at different concentrations are shown in Figure 3. It is evident that in the range of the tested concentrations, as the shear rate and the mucilage concentration increase, the shear strain rises (Figure 3a). The viscosity of the mucilage obtained from dehydrated cladodes is higher than that of the mucilage obtained from fresh cladodes.

The rheogram in Figure 3b shows that as the shear rate increases, the viscosity of the samples decreases. This means that the solutions of the mucilage extracted from fresh and dehydrated cladodes show the behavior of shear-thinning fluids, also referred to as pseudo-plastic fluids in the evaluation conditions [68]. The viscosity of these fluids decreases with increasing shear rate, which is common in polymer solutions and similar solutions of high molecular weight compounds [69]. The pseudoplastic behavior of mucilage dissolutions are supported by the flow behavior index (n < 1, see Table 2). This behavior is more pronounced when the mucilage concentration increases, which is in accordance with previously reported investigations by Contreras-Padilla et al. [17], León-Martínez et al. [37] and Medina-Torres et al. [11].

Interestingly, the mucilage obtained from dried cladodes shows higher viscosity values than the mucilage extracted from the fresh cladodes (see Figure 3b), in fact, in the entire range of concentrations evaluated, the viscosity values of the DNM were approximately twice the respective values of the FNM. This was corroborated with the observation that the consistency index (K) values were higher in the DMC (see Table 2).

At this point, it is important to mention that significant differences were observed in the calcium content of the DNM and the FNM (see Table 1). It has been reported that the presence of calcium ions caused an increase in the viscosity of the cell wall polysaccharide matrix. Likewise, an increase in the viscosity is accompanied by a rise in the calcium ions concentration [70].

Evidently, the difference in the viscosity of the FNM and the DNM will have a significant impact on the textural properties of the products to which these mucilages are added. For example, the FNM can be used in products where a moderate or low viscosity is needed to replace dairy or fats (e.g., frozen desserts) and to make edible coatings and films on minimally processed products [71,72]. In contrast, the DNM can be useful to improve textural characteristics of products and to replace egg and fats (e.g., dressings) [71].

### 3.7. Thermogravimetric Analysis

**Figure 4:**
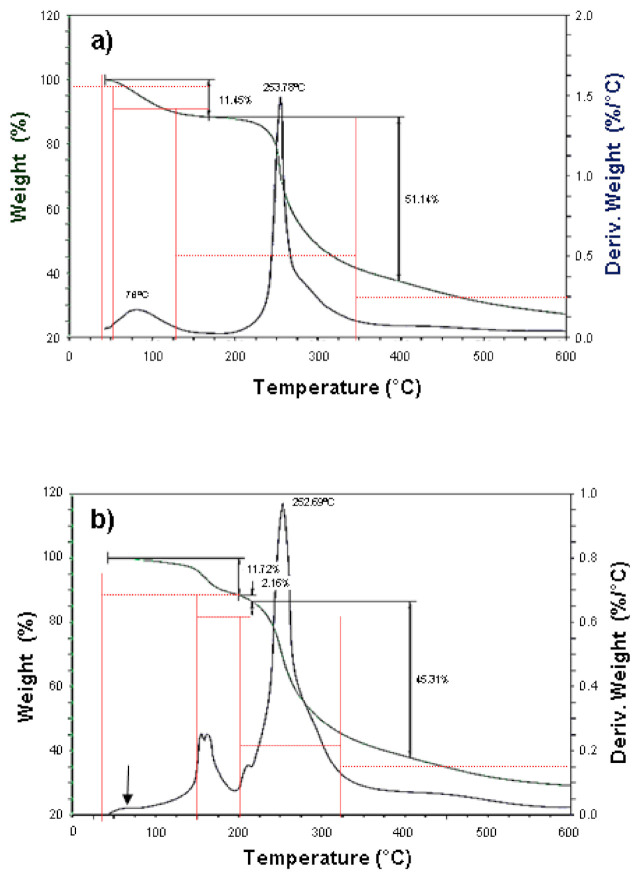
Thermogravimetric curve and derivative thermogram curve of (**a**) mucilage extracted from fresh *O. ficus indica* cladodes and (**b**) mucilage extracted from dehydrated *O. ficus indica* cladodes.

Thermogravimetric analysis (TGA) is a simple and accurate method to measure the change in weight of a sample as a function of a temperature profile. This technique is useful for determining sample decomposition, oxidation or loss of solvent or water [73]. Figure 4 shows the thermograms of the mucilage extracted from fresh (Figure 4a) and dehydrated (Figure 4b) cladodes. Table 3 provides the details of thermal behavior according to the primary thermograms and derivative thermograms for the samples. The early minor weight loss in the FNM is attributed to the removal of free water molecules (adsorbed water) and those water molecules bound to the polysaccharides contained in the mucilage [15,73,74].

Since the first weight loss in the FNM occurs over a wide range of temperatures (see Table 3), some authors have proposed that it might be associated with the loss of low molecular weight components (solvent extractable components) present in *O. ficus indica*, such as lipids, chlorophyll, phenolic components among others [54,75,76,77,78]. By contrast, this was not observed in the DNM (Figure 4b see arrow). It is likely that most of the adsorbed water and volatile compounds were eliminated during the cladode drying process prior to the extraction of the mucilage, so the elimination of these components was not detected. Interestingly, the first event in DNM was observed at a temperature range of 125 to 200 °C with two maximum temperature peaks (see Figure 4 and Table 3). Regarding this, it has been previously reported that the weight loss between 100–200 °C may be attributed to the loss of water that is hydrogen-bound and/or coordinated to mineral compounds containing calcium [79]. In the case of the present study, these maximum temperature peaks are probably due to the loss of water attached to the calcium compounds (CaCO_3_, K_2_Ca(CO_3_)_2_) identified in *O. ficus indica* mucilage [6,17].

The second event exhibited the highest mass loss in FNM and DNM (51.14 and 45.31%, respectively) in similar temperature peaks (see Table 3). These data are comparable with thermal stability observed in mucilage extracted from *Hollyhocks* seeds, *Opuntia spinulifera* and modified *Dalbergia sissoo* gum reported by other authors [77,80,81]. This event is attributed to the thermal degradation onset of the biopolymers such as branch ruptures of polysaccharides, and decomposition of pectins [15,77]. The residual mass at 600 °C, which is greater in DNM compared to FNM (see Figure 4), has been associated with the content of inorganic compounds quantified as ash in the mucilage (see Table 1) [77]. It is important to mention that the weight loss onset in samples suggests that both FNM and DNM have a good thermal stability, which indicates that FNM and DNM could be used in products that require sterilization temperatures [82].

### 3.8. Color Parameters of Mucilage Extracted from Cladodes

The color parameters (L*, a*, and b*) of the mucilage extracted from dehydrated and fresh *O. ficus indica* cladodes are shown in Table 4. Significant differences in all parameters were found between both mucilages. The FNM had a lighter color (green), than that of the DNM (green with brown tones). The ΔE = 8.33 indicates the color difference between the mucilage samples, this difference has implications for their possible applications.

In accordance with the aforementioned, the mucilage extracted from both fresh and dehydrated cladodes can be used for the development of soft capsules (softgels), for which an L* = 87.73 is accepted for capsule control (standard) [83]. On the other hand, the FNM (which had the lighter color) would be suitable for the formulation of edible films, where a light transmission rate and film transparency are required [72,84]. On the contrary, the DNM could be used as an encapsulating agent to preserve the stability of bioactive compounds and food additives, as well as to improve textural and functional properties of foods, where color is not a critical attribute [85]. The drying process caused a decrease in the L coordinate and an increase in a* and b* in DMN compared to the values of FMN. This means that drying turns the mucilage less light and green. Color modifications and visual green color loss can be attributed to the conversion of chlorophylls to pheophytins (pheophytinization), as a result of heating and pH lowering during processing [86]. In this reaction, chlorophyll is converted to pheophytin by the loss of the magnesium ion, found in the center of the porphyrin ring, which is replaced by two hydrogen ions. Consequently, the bright green color of chlorophyll turns olive-brown, which causes a negative perception in the consumer [87]. Changes in chlorophyll concentration are linearly related to the *a value, for this reason, this parameter is used as a quality indicator of thermally processed vegetables [86].

### 3.9. Texture Attributes of the Mucilage Extracted from Cladodes

Texture profile analysis (TPA) is a technique that has been widely employed for determining the textural properties of foods, although it is also used in the cosmetic and pharmaceutic industries. During a TPA test, samples are compressed twice, using a texture analyzer to provide insight into how samples behave when chewed [88]. The parameters (attributes) that may be derived from TPA are hardness (force required for a predetermined deformation), cohesiveness (strength of internal bonds in the sample), springiness (originally called elasticity, that is the rate at which the deformed sample returns to its undeformed condition after the removal of the deforming force), adhesiveness (work required to overcome the attractive forces between the surface of the sample and the surface of the probe with which the sample comes into contact), compressibility (the force per unit time required to deform the product during the first compression cycle of the probe), among others [89]. Test conditions greatly affects the results derived from a TPA. Moreover, the measured and calculated TPA parameters have a slight relationship to the same properties in Material Science and other disciplines, for this reason, TPA parameters are currently subject of study [90]. In order to preliminarily assess the possible applications of *O. ficus indica* mucilage as an additive, a TPA was carried out. The instrumental texture profile of cohesiveness, adhesiveness, and springiness for mucilage samples are presented in Table 5.

Statistically significant differences (*p* < 0.05) in cohesiveness, adhesiveness, springiness, hardness, and compressibility were detected between the mucilage obtained from fresh and dehydrated cladodes samples, which indicates that the dehydration process of cladodes prior to mucilage extraction significantly affects TPA parameters, reducing cohesiveness, adhesiveness, and springiness of the mucilage. According to an engineering approach of food processing, texture attributes are the result of structure and composition that are obtained by submitting the ingredients to a sequence of operations, which comprise a given food process [91]; specifically, cohesion between two particles and adhesion between two surfaces without material bridges is primarily attributable to Van der Waals’ interactions and electrostatic forces [92]. From this point of view, the decrease in cohesiveness and adhesiveness of DNM compared to FNM can be attributed to the loss of hydrogen bonds between water and hydroxyl groups of sugars and the carboxyl group of uronic acid contained in the mucilage. This proposal is supported as previously discussed by the reduction in the frequency of the characteristic broad band corresponding to the OH group stretching vibration and for the carboxylate group (COO^-^) (see Figure 2). It is worth mentioning that hardness and compressibility values in DNM were higher than those observed in FMN. Probably, the loss of hydrogen bonds in DNM favored the interaction between the ionized carboxylate group of uronic acid and calcium and other cations, which increases the viscosity of DNM (see Figure 3), and consequently its hardness and compressibility.

## 4. Conclusions

The development of sustainable extraction methods to obtain natural products that can be used as additives with functional properties, by using agricultural products that lack commercial value, constitutes a challenge for the food industry and requires a multidisciplinary research work. In this study, we carried out a comparative analysis of the chemical composition and physicochemical properties of the mucilage extracted from fresh and dehydrated *O. ficus indica* cladodes at late maturity stage, which are considered as a by-product of agroindustry. The yield and efficiency values obtained through the process used to extract the mucilage from the *O. ficus indica* cladodes indicate that it is a sustainable extraction method. The dehydration process of cladodes prior to mucilage extraction modified the chemical composition and physicochemical properties of the mucilage, which directly influence its possible applications. The chemical composition and physicochemical characteristics of the mucilage extracted from either fresh or dehydrated *O. ficus indica* cladodes support the great potential application of this natural polymer as a food additive and as an excipient and to obtain microstructures for the development of new novel food, pharmaceutical, and cosmetic products.

## Figures and Tables

**Table 1:** Chemical proximate analysis and calcium content in mucilage extracted from fresh and dehydrated *Opuntia ficus indica* cladodes.

Component	FNM	DNM
Moisture (%)	4.12 ± 0.02 ^a^	4.19 ± 0.02 ^a^
Fat (%)	2.11 ± 0.10 ^a^	2.13 ± 0.01 ^a^
Protein (%)	6.52 ± 0.10 ^a^	7.93 ± 0.02 ^b^
Ashes (%)	3.15 ± 0.02 ^a^	5.18 ± 0.02 ^b^
Crude fiber (%)	0.33 ± 0.01 ^a^	0.31 ± 0.01 ^a^
NFE (%)	83.77 ± 0.02 ^a^	80.26 ± 0.04 ^b^
Calcium (mg/g)	15.18 ± 0.15 ^a^	19.05 ± 0.25 ^b^

The values represent the mean ± standard deviation (SD), *n* = 5. Means in rows with different letters differ significantly (*p* ≤ 0.05). NFE = Nitrogen free extract. FNM = Mucilage extracted from fresh cladodes. DNM = Mucilage extracted from dehydrated cladodes.

**Table 2:** The Power law values of mucilage extracted from *O. ficus indica* cladodes at different mucilage concentrations.

Type of Mucilage	Concentration (% *w*/*v*)	K	*n*
FNM	1	0.0635	0.7273
	1.5	0.2517	0.5824
	2	0.8697	0.4335
	2.5	1.6245	0.3828
DNM	1	0.3854	0.5414
	1.5	0.8657	0.4927
	2	1.5288	0.4519
	2.5	2.8021	0.4171

FNM = mucilage extracted from fresh cladodes, DNM = mucilage extracted from dehydrated cladodes, K = consistency index, *n* = flow behavior index.

**Table 3:** Thermogravimetric (TGA and DTG) data of the mucilage extracted from fresh and dehydrated cladodes.

Type of Mucilage	No. of Decomposition Stage	Temperature Range °C	DTG max. °C	Weight Loss %
FNM	1	45–125	76	11.45
	2	200–350	254	51.14
DNM	1	125–200	151 and 164	11.42
	2	210–325	252	45.31

FNM = mucilage extracted from fresh cladodes, DNM = mucilage extracted from dehydrated cladodes.

**Table 4:** Color parameters of mucilage extracted from fresh and dehydrated *O. ficus indica* cladodes.

Type of Mucilage	L*	a*	b*
FNM	90.34 ± 0.23 ^a^	+18.31 ± 0.15 ^a^	+22.29 ± 0.10 ^a^
DNM	84.58 ± 0.16 ^b^	+21.21 ± 0.11 ^b^	+27.57 ± 0.10 ^b^

Values represent the mean ± standard deviation (SD), *n* = 5. Means in columns with different letters differ significantly (*p* ≤ 0.05). FNM = mucilage extracted from fresh cladodes, DNM = mucilage extracted from dehydrated cladodes.

**Table 5:** Instrumental texture attributes in mucilage extracted from fresh and dehydrated *Opuntia ficus indica* cladodes.

Type of Mucilage	Cohesiveness	Adhesiveness (J × 10^−3^)	Springiness (m)	Hardness (g)	Compressibility (g·s)
DNM	0.72 ± 0.01 ^a^	3.21 ± 0.01 ^a^	0.63 ± 0.01 ^a^	11.29 ± 0.04 ^a^	77.11 ± 0.02 ^a^
FNM	0.97 ± 0.01 ^b^	5.95 ± 0.01 ^b^	1.13 ± 0.06 ^b^	6.19 ± 0.02 ^b^	42.42 ± 0.02 ^b^

Values represent the mean ± standard deviation (SD), *n* = 5. Means in columns with different letters differ significantly (*p* ≤ 0.05). FNM = mucilage extracted from fresh cladodes, DNM = mucilage extracted from dehydrated cladodes.

## Data Availability

The data that support the findings of this study are available from the corresponding author upon reasonable request.

## References

[1] Maki-Díaz G., Peña-Valdivia C., García-Nava R., Arévalo-Galarza M., Calderón-Zavala G., Anaya-Rosales S. (2015). Physical and chemical characteristics of cactus stems (*Opuntia ficus-indica*) for exportation and domestic markets. Rev. Agro..

[2] Contreras-Padilla M., Gutiérrez-Cortez E., Valderrama-Bravo M., Rojas-Molina I., Espinosa-Arbeláez G., Suárez-Vargas R., Rodríguez-García M. (2012). Effects of drying process on the physicochemical properties of nopal cladodes at different maturity stages. Plant Foods Hum. Nutr..

[3] Kaur M., Kaur A., Sharma R. (2011). Pharmacological actions of *Opuntia ficus indica*: A Review. J. App. Pharm. Sci..

[4] Osuna-Martínez U., Reyes-Esparza J., Rodríguez-Fragoso L. (2014). Cactus (*Opuntia ficus-indica*): A review on in antioxidants propierties and potential pharmacological use in chronic diseases. Nat. Prod. Chem. Res..

[5] Tilahun Y., Welegerima G. (2018). Pharmacological potential of cactus pear (*Opuntia ficus-indica*): A review. Int. J. Pharmacogn. Phytochem. Res..

[6] Rojas-Molina I., Gutiérrez-Cortez E., Moustapha E., Rojas-Molina A., Ibarra-Alvarado C., Rivera-Muñoz E., De Real A., Aguilera-Barreiro A. (2015). Characterization of calcium compounds in *Opuntia ficus indica* as a source of calcium for human diet. J. Chem..

[7] Hernández-Becerra E., Gutiérrez-Cortez E., Del Real A., Rojas-Molina A., Rodríguez-García M., Rubio M., Quintero-García M., Rojas-Molina I. (2017). Bone mineral density, mechanical, microstructural properties and mineral content of the femur in growing rats fed with *Opuntia ficus indica* as calcium source in diet. Nutrients.

[8] Quintero-García M., Gutiérrez-Cortez E., Rojas-Molina A., Mendoza-Ávila M., Del Real A., Rubio E., Jiménez-Mendoza D., Rojas-Molina I. (2020). Calcium bioavailability of *Opuntia ficus-indica* cladodes in an ovariectomized rat model of postmenopausal bone loss. Nutrients.

[9] Mendoza-Ávila M., Gutiérrez-Cortez E., Quintero-García M., Del Real A., Rivera-Muñoz E., Ibarra-Alvarado C., Rubio E., Jiménez-Mendoza D., Rojas-Molina I. (2020). Calcium bioavailability in the soluble and insoluble fibers extracted from *Opuntia ficus indica* at different maturity stages in growing rats. Nutrients.

[10] Medina-Torres L., González-Laredo R., Gallegos-Infante J., Rocha-Guzmán N. (2008). Drying kinetics of nopal (*Opuntia ficus indica*) using three different methods and their mechanical properties. Food. Sci. Technol..

[11] Medina-Torres L., Brito-De la Fuente E., Torrestiana-Sánchez B., Katthain R. (2000). Rheological properties of the mucilage gum (*Opuntia ficus-indica*). Food Hydrocoll..

[12] Espino-Díaz M., Ornelas-Paz J., Martínez-Téllez M., Santillán C., Barbosa-Cánovas G., Zamudio-Flores P., Olivas G. (2010). Development and characterization of edible films based on mucilage of *Opuntia ficus-indica* (L.). Int. J. Food. Sci..

[13] Medina-Torres L., García-Cruz E., Calderas F., Rodríguez-Ramírez J. (2013). Microencapsulation by spray drying of gallic acid with nopal mucilage (*Opuntia ficus indica*). Food Sci. Techno. Int..

[14] Zambrano-Zaragoza M., Gutiérrez-Cortez E., Del Real A., González-Reza R., Galindo-Pérez M., Quintanar-Guerrero M. (2014). Effect of coating on polyphenol oxidase and pectin methylesterase activities. Innov. Food Sci. Emerg. Technol..

[15] Archana G., Sabina K., Babuskin S., Radhakrishnan K., Fayidh M., Babu P., Sivarajan M., Sukumar M. (2013). Preparation and characterization of mucilage polysaccharide for biomedical applications. Carbohydr. Polym..

[16] Rodríguez-González S., Martínez-Flores H., Chávez-Moreno C., Macías-Rodríguez I., Zavala-Mendoza E., Garnica-Romo M., Chacón-García L. (2014). Extraction and characterization of mucilage from wild species of *Opuntia*. J. Foods Process. Preserv..

[17] Contreras-Padilla M., Rodríguez-García E., Gutiérrez-Cortez E., Valderrama-Bravo M., Rojas-Molina I., Rivera-Muñoz E. (2016). Physicochemical and rheological characterization of *Opuntia ficus* mucilage at three different maturity stages of cladode. Eur. Polym. J..

[18] Saenz C., Sepúlveda E., Matsuhiro B. (2004). *Opuntia spp* mucilage’s: A functional component with industrial perspectives. J. Arid. Environ..

[19] AOAC (2000). Official Methods of Analysis of AOAC International.

[20] Bostan A., Seyed M.A., Farhoosh R. (2010). Optimization of hydrocolloid extraction from wild sage seed (*Salvia macrosiphon*) using response surface. Int. J. Food Prop..

[21] Bai Z., Hu X., Wang B., Hu Z., Yang X., Zao T. (2020). Optimization of shaft-seal water system of cutter suction dredger based on high efficiency centrifugal separation technology. Sep. Purif. Technol..

[22] ASTM (2012). Libro Annual de Normas ASTM. Sección 11. Agua y Tecnología Ambiental.

[23] American Association of Cereal Chemists (2000). Approved Methods.

[24] Mendoza-Avila M., Rojas-Molina I., Cornejo-Villegas M., Del Real-Lopez A., Rivera-Muñoz E., Rodríguez-García M., Gutiérrez-Cortez E. (2020). Physicochemical properties and resistant starch content of corn tortilla flours refrigerated at different storage times. Foods.

[25] Hussain N., Ishak I., Sulaiman R., Fauzi W.M., Coorey R. (2020). Influence of processing conditions on rheological properties of aqueous extract chia (*Salvia hispanica* L.) mucilage. Food Res..

[26] Gheribi R., Puchot L., Pierre V., Jaoued-Grayaa N., Mezni M., Habibi Y., Khaoula K. (2018). Development of plasticized edible films from *Opuntia ficus-indica* mucilage: A comparative study of various polyol plasticizers. Carbohydr. Polym..

[27] Sepúlveda E., Sáenz C., Aliaga E., Aceituno C. (2007). Extraction and characterization of mucilage in *Opuntia* spp.. J. Arid. Environ..

[28] Monrroy M., García E., Ríos K., Renán J. (2017). Extraction and physicochemical characterization of mucilage extracted from *Opuntia cochenillifera* (L.) Miller. J. Chem.

[29] Missaoui M., D’Antuono I., D’Imperio M., Linsalata V., Buokhchina S., Logrieco A., Cardinali A. (2020). Characterization of micronutrients, bioaccesibility and antioxidant activity of prickly pear cladodes as functional ingredient. Molecules.

[30] Ho C.H.L., Cacacea J.E., Mazza G. (2007). Extraction of lignans, proteins and carbohydrates from flaxseed meal with pressurized low polarity water. LWT Food Sci. Technol..

[31] Hernández-Urbiola M., Contreras-Padilla M., Perez-Torrero E., Hernández-Quevedo G., Rojas-Molina I., Cortes M. (2010). Study of nutritional composition of nopal (*Opuntia ficus indica* cv. Redonda) at different maturity stages. Open Nutr. J..

[32] Grosso C., Valentão P., Ferreres F., Andrade P.B. (2015). Alternative and efficient extraction methods for marine-derived compounds. Mar. Drugs.

[33] Cong Q., Chen H., Liao W., Xiao F., Wang P., Qin Y., Ding K. (2016). Structural characterization and effect on anti-angiogenic activity of a fucoidan from Sargassum fusiforme. Carbohydr. Polym..

[34] Gomez L., Alvarez C., Zhao M., Tiwari U., Curtin J., Garcia-Vaquero M., Tiwari B.K. (2020). Innovative processing strategies and technologies to obtain hydrocolloids from macroalgae for food applications. Carbohydr. Polym..

[35] Capello C., Fischer U., Hungerbuhler K. (2007). What is a Green Solvent? A comprehensive framework for the environmental assessment of solvents. Green Chem..

[36] Matos G.S., Pereira S.G., Genisheva Z.A., Gomes A.M., Teixeira J.A., Rocha C.M.R. (2021). Advances in extraction methods to recover added-value compounds from seaweeds: Sustainability and Functionality. Foods.

[37] León-Martínez F.M., Méndez -Lagunas L.L., Rodríguez-Ramírez J. (2010). Spray drying of nopal mucilage (*Opuntia ficus-indica)*: Effects on powder properties and characterization. Carbohydr. Polym..

[38] Otálora M., Carriazo J., Iturriaga L., Nazacero M., Osorio C. (2015). Microencapsulation of betalains obtained from cactus fruit (*Opuntia ficus indica*) by spray drying using cactus cladode mucilage and maltodextrin as encapsulating agents. Food Chem..

[39] Toit A., Wit M., Arno H. (2018). Cultivar and harvest month influence the nutrient content of *Opuntia* spp. cactus pear cladode mucilage extracts. Molecules.

[40] Gebresamuel N., Gebre-Mariam T. (2012). Comparative physico-chemical characterization of the mucilages of two cactus pears (*Opuntia* spp.) Obtained from Mekelle, northern Ethiopia. J. Biomater. Nanobiotechnol..

[41] Charuwat P., Boardman G., Bott C., Novak J.T. (2018). Thermal degradation of long chain fatty acids. Water Environ. Res..

[42] Majdoub H., Roudesli S., Picton L., Le Cerf D., Muller G., Grisel M. (2001). Prickly pear nopals pectin from *Opuntia ficus indica* physico-chemical study in dilute and semi-dilute solutions. Carbohyd. Polym..

[43] Goycoolea F., Cardenas A. (2003). Pectins from *Opuntia* spp.: A short review. J. Prof. Assoc. Cactus. Dev..

[44] Fiber Analysis of Animal Feed-FOSS Analytical. File:///C:/Users/USUARIO/Downloads/eBook-Fibre-analysis-of-animal-feed-GB.pdf.

[45] El-Mostafa K., Kharrassi Y., Badreddine A., Andreoletti P., Vamecq J., Kebbaj M., Latruffe N., Lizard G., Nasser B., Cherkaoui-Malki M. (2014). Nopal cactus (*Opuntia ficus-indica*) as a source of bioactive compounds for nutrition, health and disease. Molecules.

[46] Mounir B., Younes E.G., Asmaa M., Abdeljalil Z., Abdellah A. (2020). Physico-chemical changes in cladodes of Opuntia ficus-indica as a function of the growth stage and harvesting areas. J. Plant Physiol..

[47] McClements D.J. (2004). Protein-stabilized emulsions. Curr. Opin. Colloid Interface Sci..

[48] Malainine M., Dufresne A., Dupeyre D., Vignon R., Mahrouuz M. (2003). First evidence for the presence of weddellite crystallites in *Opuntia ficus indica* parenchyma. Z. Naturforsch. C J. Biosci..

[49] Perrotta V., Arambarri A. (2018). Cladodes anatomy of *Opuntia* (Cactaceae) from province of Buenos Aires (Argentina). Bol. Soc. Argent. Bot..

[50] Conde L. (1975). Anatomical comparisons of five species of *Opuntia* (Cactaceae). Missouri. Bot. Gard..

[51] Trachtenber S., Mayer A. (1982). Biophysical properties of *Opuntia ficus-indica* mucilage. Phytochemistry.

[52] Silva H., Acevedo E., Silva P. (2001). Anatomía del tejido fotosintético de diez taxa de *Opuntia* establecidos en el secano árido mediterráneo de Chile. Rev. Chil. Hist. Nat..

[53] Rani B., Kawatra A. (1994). Fibre constituents of some foods. Plant Foods Hum. Nutr..

[54] Ventura-Aguilar R.I., Bosquez-Molina E., Bautista-Baños S., Rivera-Cabrera F. (2017). Cactus stem (*Opuntia ficus-indica* Mill): Anatomy, physiology and chemical composition with emphasis on its biofunctional properties. J. Sci. Food. Agric..

[55] Bayar N., Kriaa M., Kammoun R. (2016). Extraction and characterization of three polysaccharides extracted from *Opuntia ficus indica* cladodes. I. J. Biol. Macromol..

[56] Chlup P.H., Bernard D., Stewart G.G. (2008). Disc stack centrifuge operating parameters and their impact on yeast physiology. Int. J. Biol. Macromol..

[57] Milledge J.J., Heaven S. (2011). Disc stack centrifugation separation and cell disruption of microalgae: A technical note. Env. Nat. Resour. Res..

[58] Das S., Chaudhury A. (2011). Recent advances in lipid nanoparticle formulations with solid matrix for oral drug delivery. Pharm. Sci. Tech..

[59] Quinzio C., Ayunta C., Alancay M., López de Mishima B., Iturriaga L. (2017). Physicochemical and rheological properties of mucilage extracted from *Opuntia ficus indica* (L. Miller). Comparative study with guar gum and xanthan gum. J. Food Meas. Charact..

[60] Cortés-Camargo S., Gallardo-Rivera R., Barragán-Huerta B., Dublán-García O., Román-Guerrero A., Pérez-Alonso C. (2017). Exploring the potential of mesquite gum-nopal mucilage mixtures: Physicochemical and functional properties. J. Food. Sci..

[61] Faria M., Mislaine K., Nicoletti V. (2017). Characterization of biopolymers and soy protein isolate-high-methoxyl pectin complex. Polímeros.

[62] Harnsilawat T., Pongsawatmanit R., McClements D.J. (2006). Characterization of b-lactoglobulin–sodium alginate interactions in aqueous solutions: A calorimetry, light scattering, electrophoretic mobility and solubility study. Food Hydrocoll..

[63] Bai L., Liu F., Xu X., Huan S., Gu J., McClements D. (2017). Impact of polysaccharide molecular characteristics on viscosity enhancement and depletion flocculation. J. Food Eng..

[64] Rosland S., Yusof Y., Chin N., Chang L., Ghazali H., Ghani M., Ishak I. (2020). The effect of particle size on the physical properties of arabic gum powder. J. Food Procees. Eng..

[65] Begum R., Yusof Y., Gulzarul M., Uddin B. (2017). Sctructural and functional properties of pectin extracted from jackfruit (*Artocarpus heterophyllus*) waste: Effects of drying. I. J. Food Sci. Prop..

[66] Acartürk F., Armagan C. (2009). Comparision of guar gum from different sources for the preparation of prolonged-release or colon-specific dosage forms. Pharm. Dev. Technol..

[67] Köse M.D., Bayraktar O., Heinz Ö.K., Grumezescu A.M. (2018). Application of Complex Coacervates in Controlled Delivery. Design and Development of New Nanocarriers.

[68] Rao A.M., Barbosa-Cánovas G.V. (2013). Flow and Functional Models for Rheological Properties of Fluid Foods. Rheology of Fluid, Semisolid, and Solid Foods, Principles and Applications.

[69] Bastian E.R. (2017). Fluids. Microfluidics: Modelling, Mechanics and Mathematics.

[70] Mierczyńska J., Cybulska J., Sołowiej B., Zdunek A. (2015). Effect of Ca^2+^, Fe^2+^ and Mg^2+^ on rheological properties of new food matrix made of modified cell wall polysaccharides from apple. Carbohydr. Polym..

[71] Toit A., De Wit M., Fouché H.J., Venter S.L., Hugo A. (2020). Relationship between weather conditions and the physicochemical characteristics of cladodes and mucilage from two cactus pear species. PLoS ONE.

[72] Zambrano-Zaragoza M.L., Gutiérrez-Cortez E., Del Real A., Ricardo M., González-Reza R.M., Galindo-Pérez M.J., Quintanar-Guerrero D. (2014). Fresh-cut Red Delicious apples coating using tocopherol/mucilage nanoemulsion: Effect of coating on polyphenol oxidase and pectin methylesterase activities. Food Res. Int..

[73] Zohuriaan M.J., Shokrolahi F. (2004). Thermal studies on natural and modified gums. Polym. Test..

[74] Singh S., Bothara S.B. (2014). Morphological, physico-chemical and structural characterization of mucilage isolated from the seeds of *Buchanania lanzan* Spreng. Int. J. Public Health.

[75] Manals-Cutiño E., Penedo-Medina M., Giralt-Ortega G. (2011). Análisis termogravimétrico y térmico diferencial de diferentes biomasas vegetales. Tec. Quím..

[76] Andreu L., Nuncio-Jáuregui N., Carbonell-Barrachina Á.A., Legua P., Hernández F. (2018). Antioxidant properties and chemical characterization of Spanish *Opuntia ficus-indica* Mill. cladodes and fruits. J. Sci. Food Agric..

[77] Madera-Santana T.J., Vargas-Rodríguez L., Núñez-Colín C.A., González-García F., Peña-Caballero V., Núñez-Gastélum J.A., Gallegos-Vázquez C., Rodríguez-Núñez R. (2018). Mucilage from cladodes of *Opuntia spinulifera* Salm-Dyck: Chemical, morphological, structural and thermal characterization. CyTA J. Food.

[78] Cruz-Rubio J.M., Mueller M., Loeppert R., Viernstein H., Praznik W. (2020). The Effect of Cladode drying techniques on the prebiotic potential and molecular characteristics of the mucilage extracted from *Opuntia ficus-indica* and *Opuntia joconostle*. Sci. Pharm..

[79] Bala P., Samantaray B.K., Srivastava S.K. (2000). Dehydration transformation in Ca-montmorillonite. Bull. Mater. Sci..

[80] Nowrouzi I., Mohammadi A.H., Manshad K.A. (2020). Characterization and likelihood application of extracted mucilage from *Hollyhocks* plant as a natural polymer in enhanced oil recovery process by alkali-surfactant-polymer (ASP) slug injection into sandstone oil reservoirs. J. Mol. Liq..

[81] Munir H., Shahidb M., Anjumc F., Mudgil D. (2016). Structural, thermal and rheological characterization of modified *Dalbergia sissoo* gum-A medicinal gum. Int. J. Biol. Macromol..

[82] Berovič M., Moo-Young M. (2011). Sterilization in Biotechnology. Comprehensive Biotechnology.

[83] Camelo L., Wilches-Torres A., Cárdenas-Chaparro A., Gómez J., Otálora M. (2019). Preparation and Physicochemical Characterization of softgels cross-Linked with cactus mucilage extracted from cladodes of *Opuntia Ficus-Indica*. Molecules.

[84] Gónzalez D., Luna B., Martínez-Ávila G., Rodríguez H., Avendaño V., Rojas R. (2019). Formulation and characterizationof edible films based on organic mucilage from mexican *Opuntia ficus-indica*. Coatings.

[85] Liguori G., Gentile C., Gaglio R., Perrone A., Guarcello R., Francesa N., Fretto S., Inglese P., Settanni L. (2020). Effect of addition of *Opuntia ficus-indica* mucilage on the biological leavening physical, nutritional, antioxidant and sensory aspects of bread. J. Biosci. Bioeng..

[86] Steet J., Tong C. (1996). Quantification of color change resulting from pheophytinization and nonenzymatic browning reactions in thermally processed green peas. Agric. Food Chem..

[87] Yilmaz C., Gökmen V., Caballero B., Finglas P.M., Toldrá F. (2016). Chlorophyll. Encyclopedia of Food and Health.

[88] Tai A., Bianchini R., Tachowicz J. (2014). Texture analysis of cosmetic, pharmaceutical raw materials and formulations. Int. J. Cosmet. Sci..

[89] Nishinari K., Kohyama K., Kumagai H., Funami T., Bourne M.C. (2013). Parameters of texture profile analysis. Food Sci. Technol. Res..

[90] Pelleg M. (2019). The instrumental texture profile analysis revisited. J. Texture Stud..

[91] Di Monaco R., Cavella S., Masi P. (2008). Predicting sensory cohesiveness, hardness and springiness of solid foods from instrumental measurements. J. Texture Stud..

[92] Adhikari B., Howes T., Bhandari B.R., Truong V. (2001). Stickiness in foods: A review of mechanisms and test methods. Int. J. Food Sci. Technol..

